# MR photography of 3D-MR images

**DOI:** 10.1186/1532-429X-18-S1-P33

**Published:** 2016-01-27

**Authors:** Robert Jablonowski, Einar Heiberg, Jane Tufvesson, Henrik Engblom, Anthony H Aletras, Håkan Arheden, Marcus Carlsson

**Affiliations:** 1 Dept. of Clinical Physiology, Skåne University Hospital in Lund, Lund University, Lund, Sweden; 2 Dept. of Biomedical Engineering, Faculty of Engineering, Lund University, Lund, Sweden, Lund, Sweden; 3 Laboratory of Medical Informatics, School of Medicine, Aristotle University of Thessaloniki, Thessaloniki, Greece

## Background

Late gadolinium enhancement MRI (LGE-MRI) has become the gold standard for assessing myocardial infarction size. In experimental infarct validation studies the left ventricle is often sliced into thin slices and subsequently stained with TTC as a gold standard for infarct quantification. There is no single imaging slice in a 3D-MR data stack that exactly represents the photographed TTC-stained slice or whole slice microscopy as the photograph is of a surface. Therefore, we developed a method for post-processing 3D-MR data to depict the exact same surface as seen on a traditionally photographed myocardial slice.

## Methods

The image analysis software Segment [1] v1.9 (http://segment.heiberg.se) was used as a platform for implementing a script enabling MR-photography. The post-processing method was validated on *ex-vivo* contrast enhanced images rendered from an experimental chronic occlusion-reperfusion pig model (n = 8). In this model the hearts were explanted after seven days of reperfusion and the left ventricle sliced into 5 mm thin slices. The slices were then stained with TTC and subsequently imaged on 1.5T Philips Achieva scanner using a high resolution T1 weighed MRI sequence (TE = 3.2 ms, TE = 2 ms, α = 70*°*, isotropic resolution 0.5 mm). Using a manually defined threshold air was nulled for every pixel in the output image and the intensity one pixel below the detected tissue surface was taken. The purpose of taking one pixel below was to remove partial volume effects in the transition between tissue and air. The image yielded from the post-processing was compared quantitatively against photographed TTC-stained slices and visually against a slice from the middle of the acquired 3D-MR volume. The hyperenhanced area on MRI was quantified using a semi-automatic weighted algorithm [2] with manual corrections and the TTC images were manually delineated.

## Results

**Figure 1:**
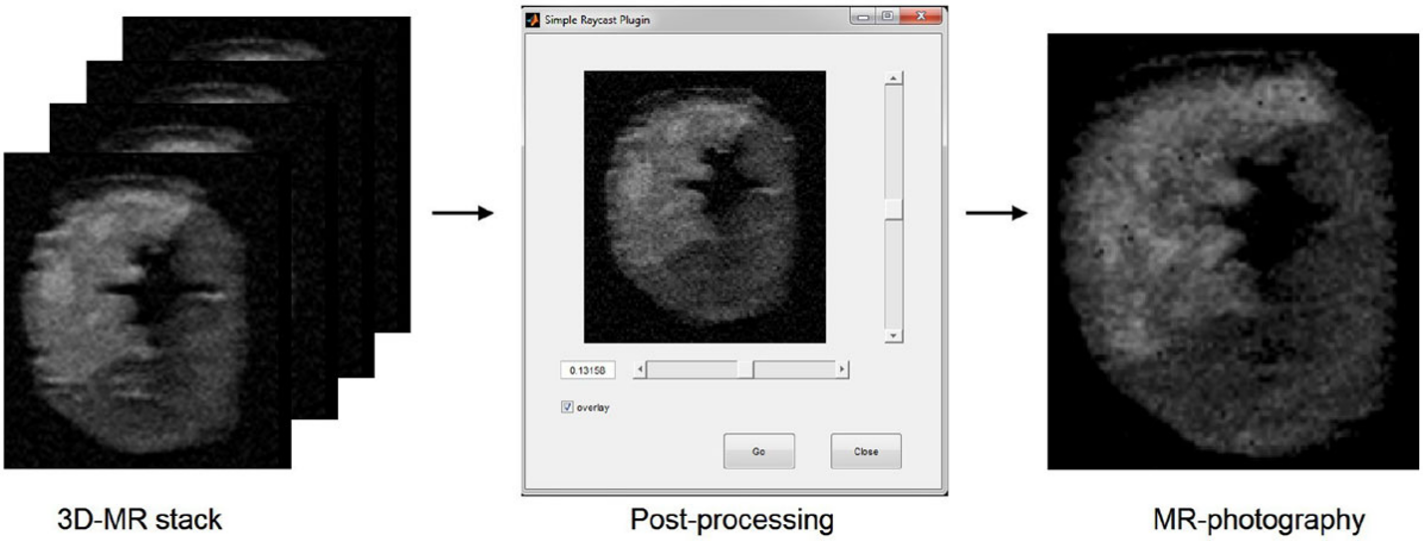
**The process of creating MR-photography**. The 3D-MR stack is loaded into the software Segment and the post-processing script is ran which enables the user to toggle, using the horizontal bar, to move down pixel-by-pixel in the image. The final processed image shows the outermost pixel of the 3D-stack, thus creating MR-photography.

**Figure 2:**
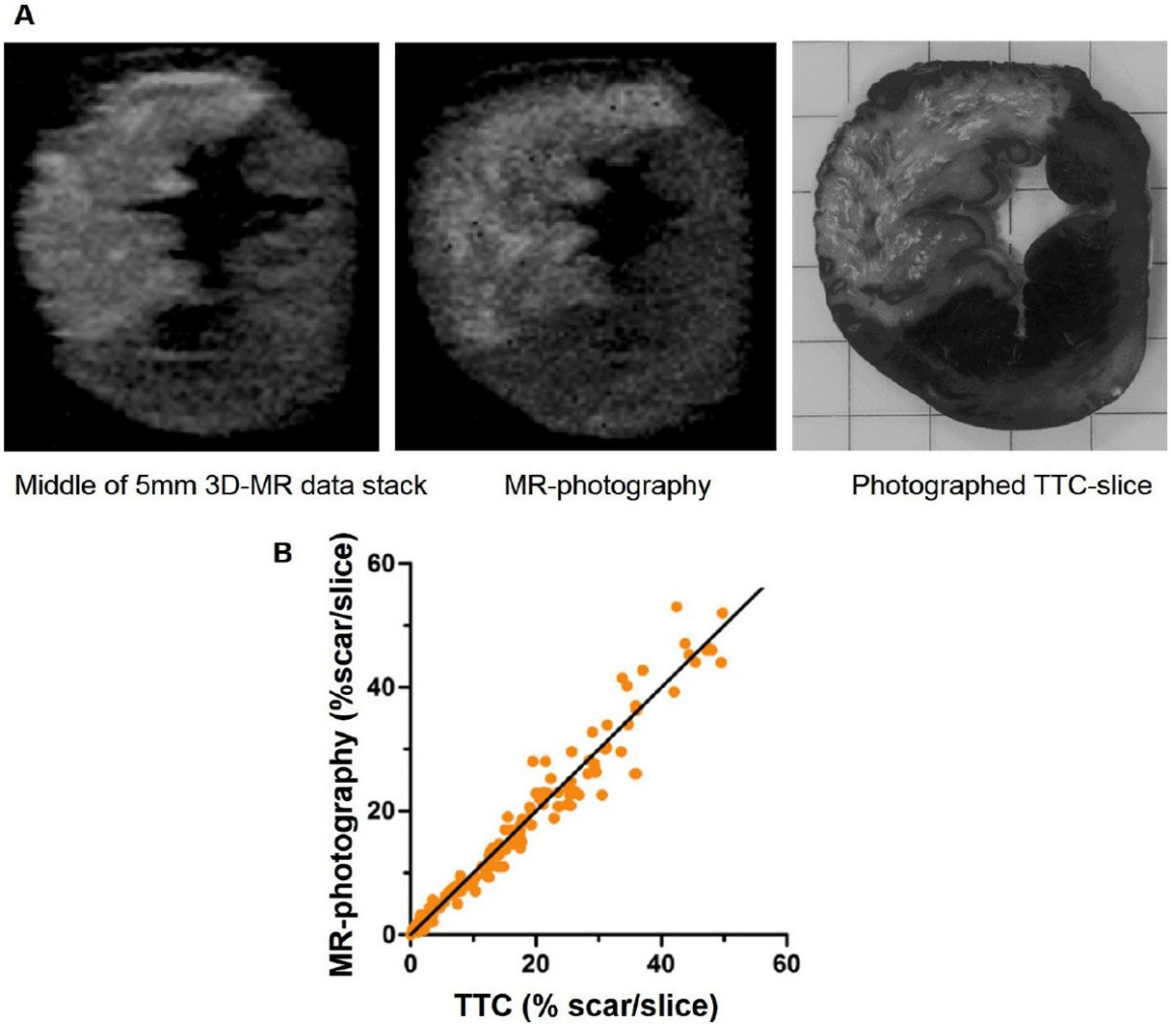
**A) Images from a 5 mm thick slice of an experimentally induced myocardial infarction imaged using contrast-enhanced MRI**. To the left, a slice from the middle of the 3D-stack, in the middle a post-processed MR-photography and to the right, the corresponding TTC-stained slice (shown in black and white). The hyperenhanced area on the post-processed MR image corresponds well with the pale necrotic area on the TTC-image, whilst the image from the middle of the stack differs. B) Scatterplot between MR-photography and corresponding TTC-stained slices.

Figure 1 shows the steps from acquired 3D-MR stack to post-processing and finally visualizing the uppermost surface of the image slab. Figure 2A shows a visual comparison of a slice in the middle of a 5 mm 3D-MR stack, MR-photography of the uppermost surface of the slice and corresponding TTC-stained slice. The hyperenhanced area on MR-photography corresponds well to the photographed TTC-slice but differs from the slice from the middle of the 3D-volume. Figure 2B shows correlation between MR-photography and corresponding TTC-slice.

## Conclusions

We have demonstrated a novel post-processing method that can be used to accurately visualize a surface from a 3D-MRI stack, thus creating MR-photography. One application demonstrated in this abstract is the comparison of traditional photographed histochemical stained tissue slices and CMR of the same tissue. The proposed methodology adds value since the infarct is heterogeneous through the imaged tissue and this will affect quantitative comparisons.
